# Rethinking Risk Assessments in a Borderline Personality Disorder Unit: Patient and Staff Perspectives

**DOI:** 10.7759/cureus.13557

**Published:** 2021-02-25

**Authors:** Owen A Crawford, Tahir S Khan, Jorge Zimbron

**Affiliations:** 1 Springbank Ward, Fulbourn Hospital, Cambridgeshire and Peterborough NHS Foundation Trust, Cambridge, GBR; 2 School of Clinical Medicine, University of Cambridge, Cambridge, GBR

**Keywords:** patient-centered care, psychiatry, mental health, risk assessment, positive risk taking, risk prediction, personality disorder, borderline personality disorder, emotionally unstable personality disorder, shared decision-making

## Abstract

Background

The present study was undertaken in a borderline personality disorder unit in Cambridge, UK. Our aim was to evaluate patient and staff perspectives on the current risk assessment procedure and to assemble opinions on a proposed change to this procedure.

Methodology

Structured interviews were conducted with patients and risk-assessing staff. Likert-scale and open questions were asked to gather both quantitative and qualitative data on both the preexisting risk assessment procedure and the proposed change to the procedure. The qualitative data was assembled into key themes.

Results

Patients and staff were moderately satisfied with the current risk assessment process, with patients scoring it an average of 2.75 out of 5 and staff scoring it 2.5 out of 5. Six key themes emerged as relevant to the process for both staff and patients: holistic approach, autonomy and freedom, responsibility, staff-patient relationship, time taken, and chance for reflection. One theme, “triggering negativity,” emerged among patients only, while a theme exploring ideas about risk emerged only among staff.

Conclusion

Our study highlights the need to introduce a new risk assessment procedure that grants patients more freedom and responsibility and encourages staff to individualize the process for each patient by taking a holistic approach. This would cultivate a ward environment that is less risk-averse and more recovery-oriented.

## Introduction

Borderline personality disorder (BPD) affects approximately 1.7% of the general population and between 15% and 28% of patients in psychiatric outpatient clinics and hospitals [1]. It is a heterogeneous disorder characterized by multiple features that can be grouped into the psychopathological categories of affective dysregulation, behavioral dysregulation, and disturbed relatedness [2]. With regards to patient safety, clinical teams are most concerned about frequent self-harm and the 50-fold increased risk of completed suicide compared to the general population [3].

To control the risks to patients and the public, mental health providers globally are often defensive in their approach [4,5]. A key component of this approach is the risk assessment, which aims to identify individuals at high risk of harm to self or others, justifying the temporary deprivation of certain liberties in order to prevent any harmful activity. A standard risk assessment for leave from the ward is widely used and is similar across the National Health Service in the UK [6]. Patients who are not detained on a section of the Mental Health Act are said to be “informal.” These patients are nevertheless required to have a conversation with a member of nursing staff when they would like to spend some time away from the ward to determine their mental state and if there are any current plans to abscond, self-harm, or attempt suicide. A checklist and summary are saved by the member of staff, which also records the patient’s recent adherence to the ward’s activities. Based on the clinical judgement of the nurse, the patient is either allowed to leave or advised that they are not safe to leave the ward at the present time.

Despite widespread use, there is limited evidence in support of the efficacy of risk assessments in reducing the risk of self-harm, suicide, or violence [7]. In addition, risk scales have insufficient evidence to support their use for predicting suicide or self-harm [8-11], and thus their utilization in the long-term management of self-harm is not recommended by the UK’s National Institute for Health and Care Excellence [12]. The risk assessment procedure leads staff to believe they are assessing risk when the evidence shows they cannot, leading to potentially dangerous false reassurance of a patient’s future safety, as well as overly defensive and coercive practices [9]. As the nature of these risks differs between patients, researchers have stressed the importance of involving the patient to estimate their individual risks for self-harm and suicide [13]. Moreover, it has been argued that a restrictive process that denies patients with mental illness the “dignity of risk” prevents them from learning through a process of trial and error, a freedom that is afforded to other members of society [14].

As part of a wider quality improvement project, we devised a new “leave protocol” that removed the need for a compulsory formal assessment of informal patients, instead giving them the freedom to choose if they wanted to talk to a member of staff before leaving the ward. The aim of this study was to understand staff and patient views on both the current leave risk assessment and the new proposed leave protocol to assess the potential impact of these changes on the main stakeholders. While some studies, to date, have looked at the views of mental health staff on risk assessments [4,15,16], none have included patients with personality disorders. Thus, we sought to add to the current literature by investigating and comparing the views of both of these parties on risk assessments.

## Materials and methods

Study setting

This study was undertaken at Springbank Ward, a 12-bed specialist ward in Cambridge, UK, for 18-65-year-old female patients with severe BPD. Patients are eligible for admission when self-destructive behavior continues to raise significant concern, despite mainstream interventions such as acute psychiatric admissions and community services. The average patient stay is approximately one year, which gives time for two six-month cycles of dialectical behavioral therapy (DBT), the primary treatment modality used for patients with BPD.

**Figure 1:**
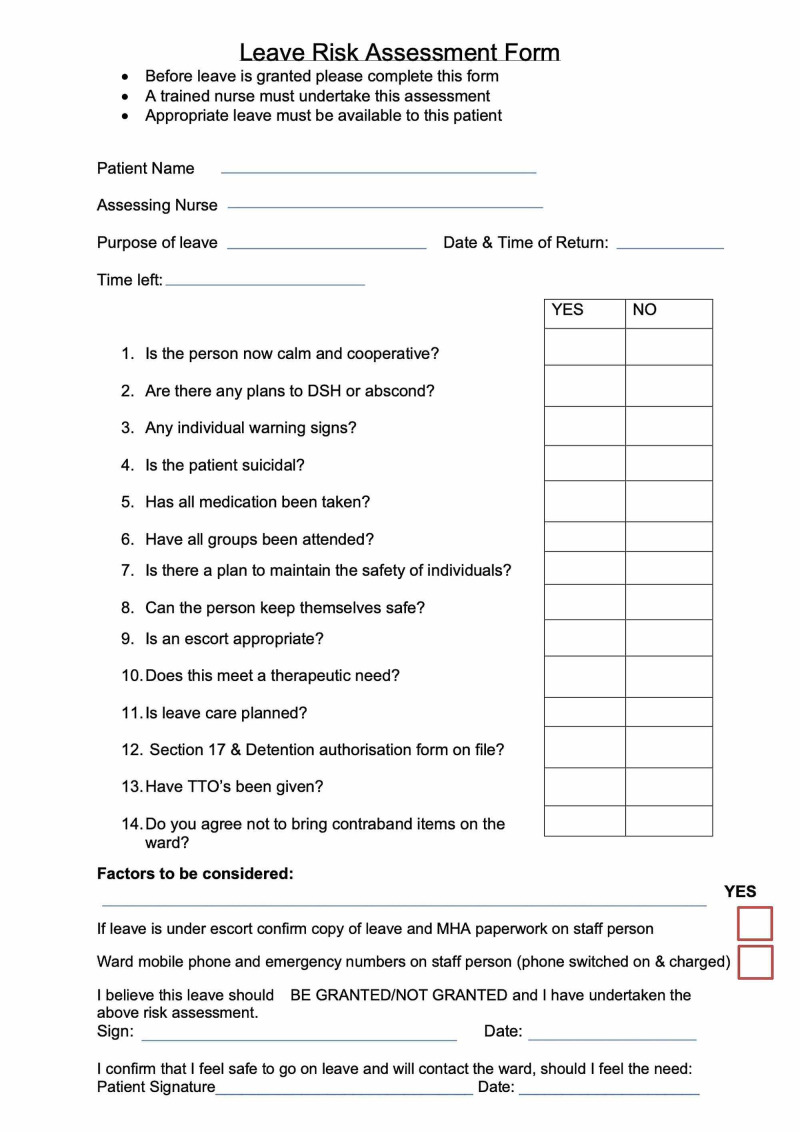
Current leave risk assessment form at Springbank Ward, Cambridge. DSH, deliberate self-harm; MHA, Mental Health Act; TTO, to take out (medications)

The ethos of Springbank Ward is based on an understanding that patients are capacitous adults who can make their own life decisions and will be discharged into the community after their admission to live relatively independently. In line with this, the ward adopts a framework of practice in which patients are encouraged to engage with treatment decisions and be as autonomous as possible. This includes the frequent granting of leave from the ward. The standard risk assessment process for granting leave, as described in the introduction, is a routine practice in the ward (Figure 1). However, this is perceived by staff as a historical remnant of the ward’s more restrictive past.

Participants

Participants included members of staff who had previously conducted leave risk assessments in Springbank Ward, as well as patients, all of whom were selected using opportunity sampling. All participants gave written informed consent to participate in the study. Interviews were held individually by OC and TK between October 2, 2019 and October 11, 2019. The ward manager and ward psychiatrist gave their approval for the project.

Data collection

**Figure 2:**
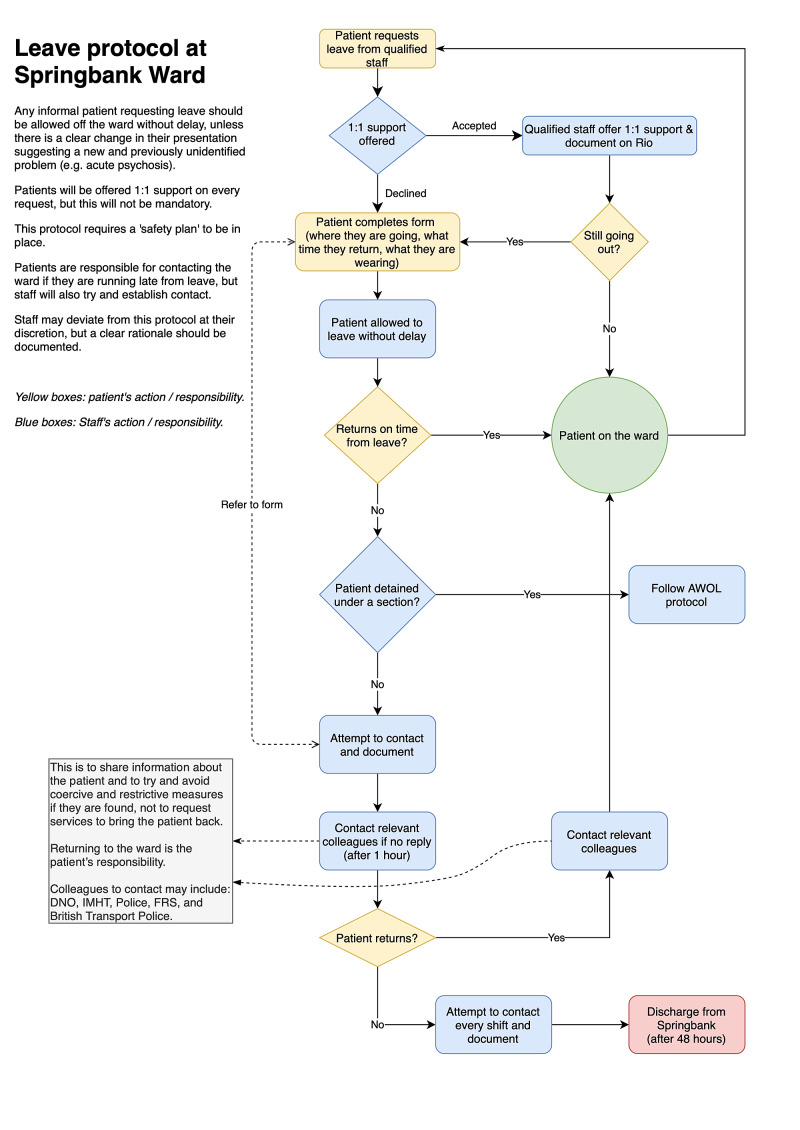
Proposed new leave protocol at Springbank Ward, Cambridge.

Likert-scale questions and open questions were devised by OC and TK, and then approved by JZ. The first set of questions sought to evaluate the current risk assessment procedure, and the second set aimed to evaluate the new leave protocol. In between the sets, a paragraph describing the new leave protocol (Figure 2) and its rationale was read out to each participant. A detailed outline of the interview structure can be found in the appendices. Interviews were held in a quiet unoccupied room in Springbank Ward to minimize the risk of responses being influenced by others. We used a structured approach in which the interviewer only asked the predetermined questions. This method was used to ensure that participant responses were focused on the process of risk assessment rather than other aspects of ward activity. Each participant’s answers to the quantitative questions were recorded, and responses to the open questions were transcribed live by the interviewer.

Data analysis

Quantitative data were collated into bar charts to visually demonstrate the distribution of answers to questions and how they compared between staff and patients. Statistical comparisons between the two groups were not calculated because of the small number of participants.

For the qualitative section, each phrase, sentence, or paragraph was individually assigned a code that best described its content, and codes were grouped together into pertinent themes. This process was initially performed independently by OC and TK. Following this, OC and TK discussed and reached a consensus on the response codes and the underlying themes.

## Results

A total of 10 members of staff and eight patients were interviewed. Of the staff members who performed risk assessments, one was an occupational therapist and nine had a nursing background. One patient and two staff members were not available during the interviewing period. Participant demographics are shown in Table 1.

**Table 1:** Participant demographics.

Participant group	Number interviewed	% Female	Mean age (range)	% White British
Patients	8	100	25.6 (18-40)	100
Staff	10	70	35.7 (26-52)	70

Quantitative results

**Figure 3:**
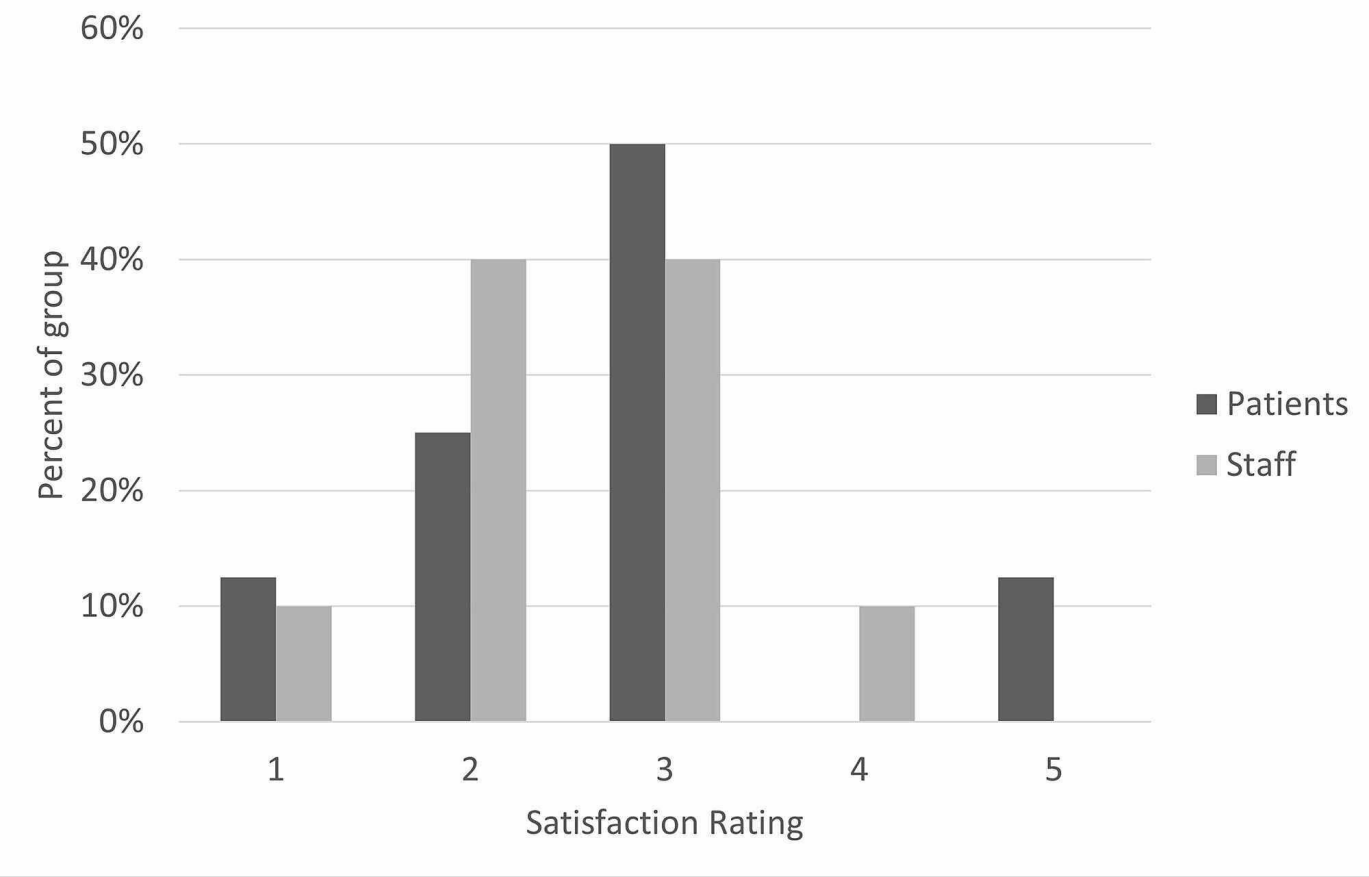
Staff and patient responses to the question, “How satisfied are you with the current risk assessment process on a scale from 1 to 5 where 5 is the most satisfied you could be?”

**Figure 4:**
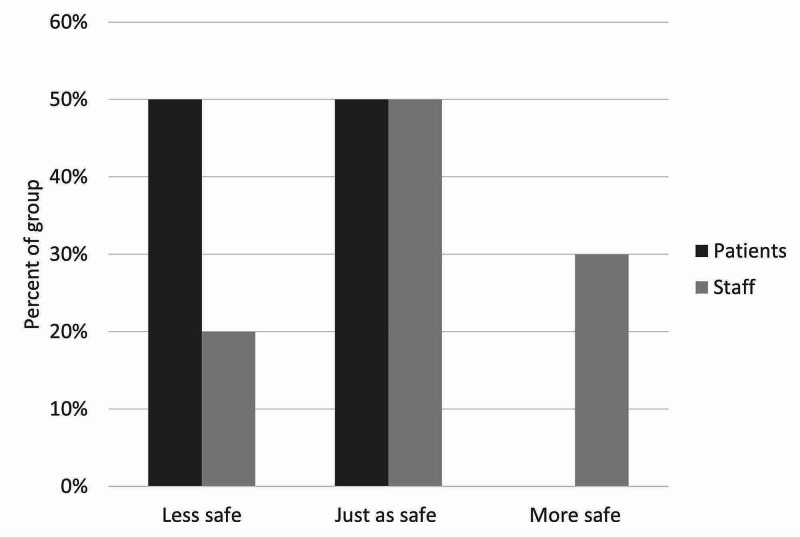
Staff and patient responses to the question, “Does this process sound more safe, less safe, or just as safe as the current process?”

**Figure 5:**
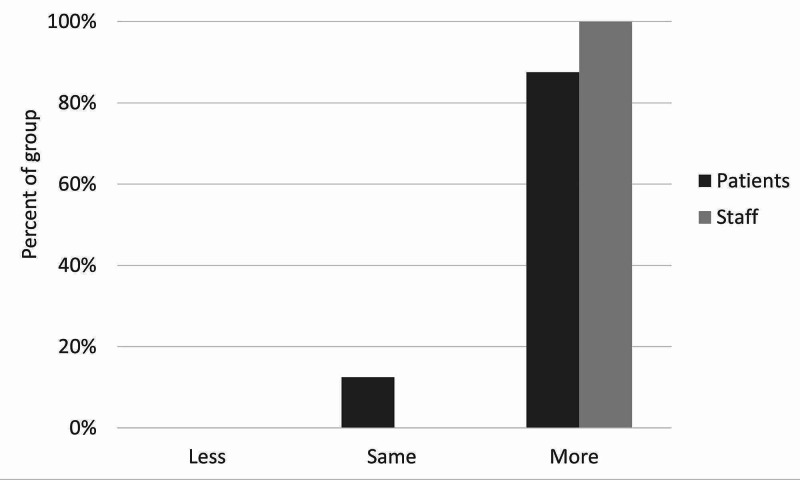
Staff and patient responses to the question, “Does it promote more patient independence, less independence, or the same?”

**Figure 6:**
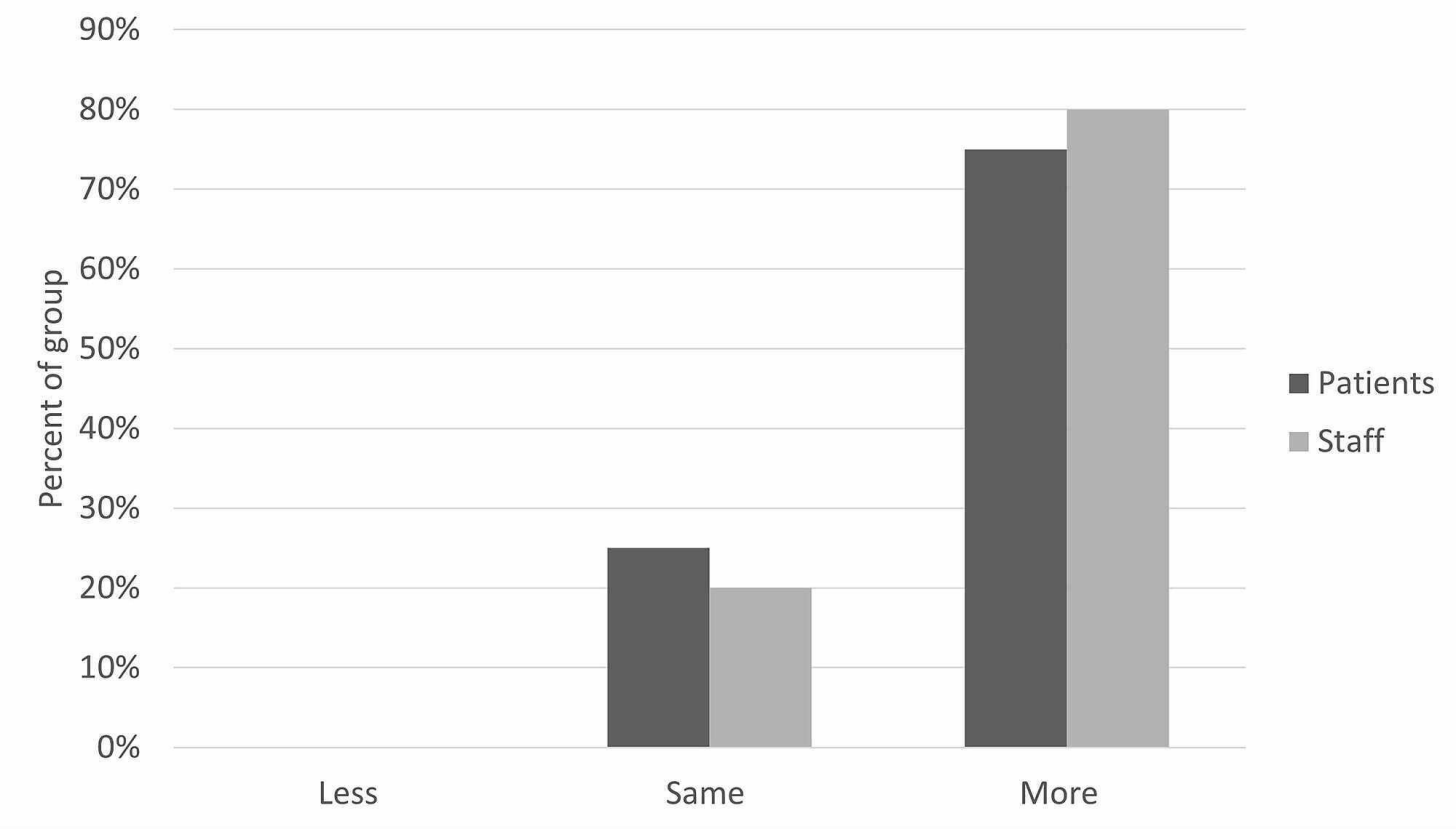
Staff and patient responses to the question, “Do you think patients would feel more responsible for their own health, less responsible, or the same?”

**Figure 7:**
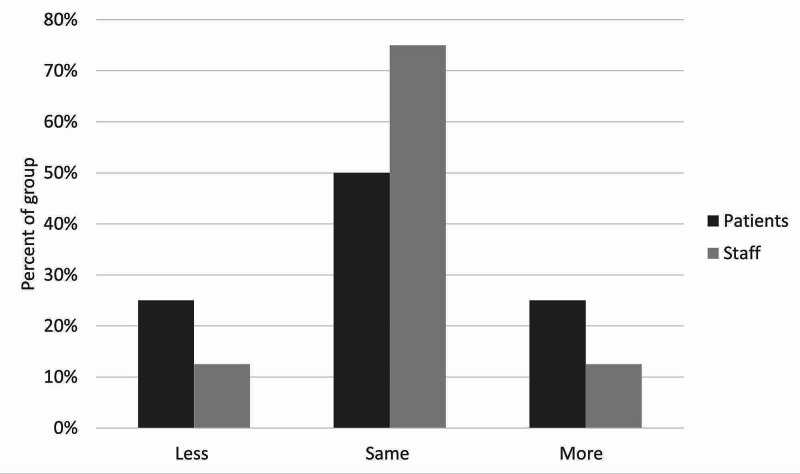
Staff and patient responses to the question, “Do you think patients would be more likely to engage in risky behaviours, less likely, or the same?”

**Figure 8:**
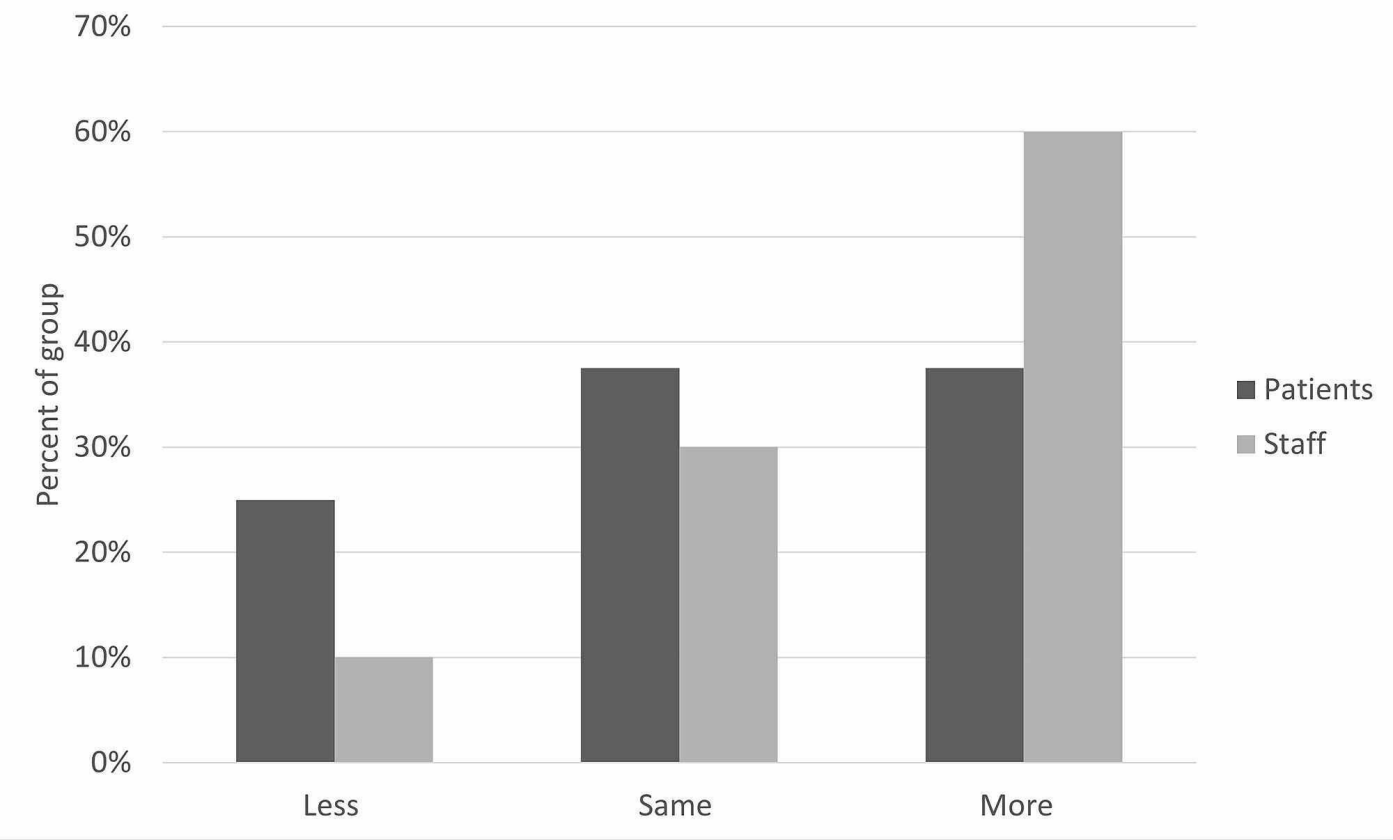
Staff and patient responses to the question, “Do you think patients would seek help from staff more, less, or the same?”

**Figure 9:**
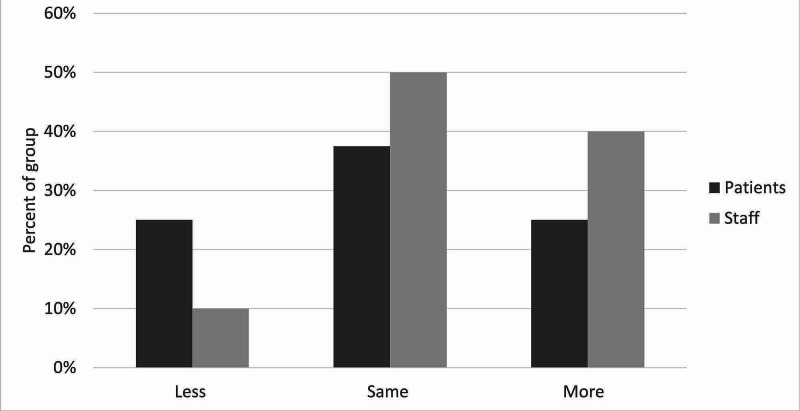
Staff and patient responses to the question, “Do you think patients would listen to staff more, less, or the same?”

There was moderate satisfaction with the current leave risk assessment for both patients and staff. The mean score out of 5 was 2.5 for staff and 2.75 for patients (figure 3). Only one patient and one staff member scored higher than 3. With regards to the new leave protocol, staff perceived it to be safer than patients did, with no patients feeling that the new procedure would be safer than the old one. With the exception of one patient, all patients and staff thought that the new leave protocol would promote more independence among patients. Most patients and staff also believed the proposed procedure would make patients feel more responsible for their health. There was more diversity of opinion in response to the question on risky behaviors, although most patients and staff thought there would be no change in their incidence. Similarly, patients and staff had mixed opinions on the likelihood of patients seeking help under the new procedure, with staff being more positive about this than patients, who remained generally neutral. Finally, the majority opinion of both patients and staff was that the new protocol would bring no change in the amount that patients listen to staff, although staff were once again more optimistic than patients on this point. These results are illustrated in Figures 3-9.

Qualitative results

Holistic Approach

The nine members of staff (90%) and five patients (63%) who spoke on this theme expressed very similar sentiments. Staff referred to the current risk assessment form as “too tick-boxey” (Staff 4), “more of a checklist, not a true assessment of risk” (Staff 10), which “can lead to complacency” (Staff 1) about the quality of their risk evaluation. Patients sensed that this made the risk assessment process too narrow and “clinical” (Patient 3). They wanted the risk assessment procedure to become more holistic and person-centered.

The suggested ways by which this would be achieved varied between participants. Staff emphasized the importance of contextual factors to determine patient risk, including how they have been feeling so far that day and how stable their relationships are at the time: “Given the emotional reactivity in the service user, it’s very much the situations rather than the individual that presents the actual risk” (Staff 2). While some patients simply wanted a catch-up with the nurse, others also wanted contextual features to be taken into account: “how the patient’s been the entire day, if they’ve had any incidents (...) and also how we’re doing in ourselves from our own viewpoint” (Patient 3). Both patients and staff generally concurred in advocating for a risk assessment that puts greater emphasis on a person-centered conversation with each patient when they ask to leave the ward.

In order to overcome the narrow view that a tick-box exercise affords, some staff have a meaningful free-flowing conversation with each patient, using the form as a guide so that all bases are covered. Others do without the form altogether, having a personalized conversation with each patient and filling in the form afterwards. Given this, Staff 8 pointed out that the risk assessment procedure does not need changing, and that the emphasis should instead be on training staff to individualize each conversation. Similarly, some patients acknowledged that certain staff make an effort to make the process more holistic: “Staff might talk more than others. One would tick tick tick yes go. One would sort of say, ‘are you okay, did you have a bad day yesterday, are you feeling okay going out today, do you want to talk’” (Patient 7). Although appreciative of some staff going above and beyond, the heterogeneity of approaches made it obvious to patients that the holistic approach used by some staff could be replicated across the board to make the process more consistently patient-centered.

Two patients went a step further, suggesting that the risk assessment procedure should be conducted based on an *a priori* evaluation of their current risk of suicidal and self-harming behaviors. Both patients said that only those with recent adverse events, for example, “when someone has had a really bad day or really bad news” (Patient 8), should have to go through a formal checklist with a nurse because of the higher perceived risk of harmful behaviors. If the patient is deemed to be at low risk at the point of wanting to go out, based on similar contextual features, then the only questions that would need to be asked are “are you okay, when will you be back” (Patient 8).

Autonomy and Freedom

A total of six (75%) patients and nine (90%) members of staff recognized autonomy and freedom as being factors relevant to the risk assessment procedure. These individuals highlighted that, as most patients are informal, they have the legal freedom to leave the ward whenever they may choose to do so, irrespective of the outcome of the risk assessment. Therefore, many felt that patient autonomy would not be affected by the change: “I don't see particularly how it’s going to change independence within the service user” (Staff 2). However, their views differed on whether patients should be given autonomy in practice.

The majority of patients felt that the current risk assessment procedure removes the decision to leave the ward and places it in the staffs’ hands: “Sometimes I might be struggling a lot but what I need is some time to myself. Not to do anything, just to get space. But sometimes in a formal risk assessment it can then be deemed as sort of risky and they sort of query whether you should be going out or not” (Patient 1). This group of patients had positive views about the new leave protocol, in that it gives them more autonomy and recognizes that they themselves are the best judge of what they need when they are distressed: “It might be that I might be distressed. But then going out on leave and going on a walk, around Tesco, around the grounds, it might benefit me, switch me from an emotional mind, chill me out, as opposed to keeping you on the ward, and you become this cold room of emotions where you also feel trapped as well” (Patient 7).

Furthermore, several members of staff pointed out that giving patients more freedom “normalizes being out in the community” (Staff 7), and various patients agreed with this view: “But also in the community it’s up to you whether you go out or not and it’s up to you whether you use risky behaviors” (Patient 1). These individuals emphasized that the ward ethos is about “providing opportunity for choice, and ownership of the choices made” (Staff 6), and the ultimate aim of the treatment program at Springbank is to re-assimilate the patients into the community. Therefore, they believed that encouraging patient autonomy and increasing freedom through positive risk-taking is a change to be welcomed.

However, a few patients pointed to the dangers of too much freedom being afforded to patients if the risk assessment process is removed. They felt that incidents in the ward would increase because “if someone is feeling dangerous then there’s nothing to stop them from acting” (Patient 2). They also alluded to the possibility that more incidents could lead to more patients being detained under the Mental Health Act sections, leading to even less freedom and autonomy for patients than they currently have. Staff 8 also viewed the proposed change negatively, saying that the patients may feel as if “they’d be on their own” as a result of giving them too much autonomy, and they may not be best placed to judge when they require help “because in the situations that have been most unsafe, the ladies would have said no” to a conversation.

Responsibility

Overall, seven (88%) patients and all 10 members of staff spoke about responsibility, making this the major theme of the study. Many staff felt that the current procedure gives mixed messages to patients in terms of whether the responsibility is on them: “On the one hand you’re saying you’re responsible for yourself, and suddenly there’s this barrier where you take back responsibility and decision-making. It’s nonsensical” (Staff 3). As an example, Staff 10 highlighted that, under the current procedure, patients are allowed to leave the ward without a risk assessment if it is for a cigarette: “What’s the difference between a piece of paper going off the ward for 15 minutes or 3 days?” Additionally, some staff had a practical concern regarding the legal responsibility for incidents, feeling that the current process assumes too much responsibility for patient behavior that cannot be predicted: “I also feel like it indemnifies the hospital or nurse should that person die. We’ve got a form saying she won’t self-harm but she’s dead, so I don’t know, I have issues with that” (Staff 6).

When they considered the proposed risk assessment procedure, some members of staff felt that increasing patient responsibility for assessing their own risk would benefit them “because they have to think about whether they feel at risk or not. And learn to communicate that. And help them to understand their emotions more if they’ve got to talk about it more” (Staff 9). Additionally, some members of staff stated that this change “fits with the idea of patients being in the community and having to ask for help when they need it, not being constantly monitored and asked by staff” (Staff 5). A few patients agreed with this view: “In the community, no one is going to risk assess me at home, so I need to be able to reflect on how I am and make a decision from that” (Patient 1). Moreover, some staff pointed out that the ethos of Springbank Ward is centered on positive risk taking, and in the past when more responsibility has been given to patients, incidents in the ward have decreased: “In the past there was a reduction in incidents on the ward when the patients stopped being restrained” (Staff 5). Staff foresaw a similar positive effect occurring from the proposed changes to the leave risk assessment procedure.

However, some members of staff felt that “women will fly under the radar” (Staff 8) in the new leave protocol, and that, as staff are trained to assess risk, responsibility should be on them to do so: “That would be up to the nurse to assess that risk and make that clinical decision whether to let them off the ward or not” (Staff 1). Interestingly, some members of staff felt that the patients want the responsibility to be on staff, and so if that desire is not met and responsibility is instead placed on themselves, it would have a negative effect: “I think we will see a peak in risky behaviors before it comes back down. Just because of the nature of the client group, and them wanting the responsibility to be on the staff rather than themselves” (Staff 10).

This perception contrasts with what we found in our patient interviews; the majority felt that responsibility should lie with themselves so that they can assess and act upon their own risk, and staff should only be responsible for providing support for those who seek help: “If it’s not a wise decision then you know that and you’re making that decision, and you can choose to do what you feel is right in that time, and if you choose not to discuss that with staff, then you’ve got to accept that and take responsibility for it” (Patient 8). Nevertheless, some patients felt that, while they are ultimately responsible for keeping themselves safe, staff should also be able to recognize when they require help and offer it. They pointed out that the reason they are not in the community is that they are not always able to assess their own risk, and thus “staff should share responsibility, because when you’re in that dangerous mindset you don’t have the ability to think so shouldn’t have full responsibility” (Patient 4).

Ultimately, most members of staff agreed that a shared-responsibility approach may be most beneficial: “Even though it might be quite nerve wracking to start with and there may be a peak in behaviors, it’s up to us and the service user to collaboratively work together and teach them the skills to manage that while off the ward” (Staff 10). In addition, Staff 5 suggested a tapered responsibility approach, where patients are given more responsibility as they spend more time in the ward: “I think that when patients are new, there should be more of a conversation about risk assessments, as they haven’t built up trust and so if they feel completely free that may be risky. New patients could come and abuse the freedom as they don’t have a relationship with staff yet.”

Staff-patient Relationship

This was another major theme, with nine (90%) members of staff and seven (88%) patients exploring it. Within this theme, there were three subsections: patient openness with staff, risk assessments as one-to-one time, and reducing conflict. With regards to openness, the general consensus among patients was that they can easily choose to be dishonest if that is necessary to be allowed off the ward: “On any risk assessments (...) it’s easy to lie” (Patient 3). Staff were aware of this, explaining that the current risk assessment process sometimes prevents open and honest conversations from taking place because the paper-based checklist often becomes the focus of attention. Over time, patients have picked up on what answers are required of them to maximize their chances of leaving the ward, resulting in dishonesty: “They still lie (...) so they can leave” (Staff 4). On the other hand, some staff gave a lot of credit to patients, saying, “they’re very good at letting us know when they’re not feeling safe” (Staff 10). Others suggested that the quality of the staff-patient relationship is actually dependent on how much time the patient has been on the ward because trust can only develop over time. This echoed Staff 5’s sentiments about having a tapered approach to patient responsibility on the ward. Additionally, openness was reported to vary between specific staff-patient relationships.

Several patients thought that the proposed changes to the process would not remove the need to lie. However, given that the conversation would become optional in the new risk assessment process, and staff members would have no right to prevent leave from the ward, other patients saw the change as encouraging more open and honest conversations: “It’s the opportunity to chat without the threat of them then not letting you out” (Patient 1). A few members of staff concurred, with Staff 3 proposing that patients would become more honest with staff if they were expected to take responsibility for their well-being through opting to talk rather than a conversation being expected of them.

Contrary to most responses, Staff 8 took the viewpoint that the current risk assessment procedure actually encourages more honesty and openness from the patient, whereas a free flowing conversation might miss some key aspects of the patient’s presentation: “I think that often you don’t get that upfront conversation with people without having that risk assessment.” According to this member of staff, patients are likely to refuse a conversation before leaving the ward under the proposed changes to the process, resulting in even less communication than at present. This idea was echoed by Staff 6, who said that “opting not to have a discussion (...) would not equip us with the knowledge of the patient’s intentions.”

Another sub-theme of the staff-patient relationship was patients’ alternative use of risk assessments as one-to-one time, which is the utilization of staff’s time for a private conversation when the patient has no intention of going out. Patients know that asking for a risk assessment ensures they will promptly have the desired one-to-one time with a member of staff, “using the time to express thoughts, feelings and internal state, because they know that time is going to be guaranteed” (Staff 2). Some saw the proposed changes to the risk assessment procedure as positive: “This will (...) encourage patients to approach staff when they need a one to one, not just using a risk assessment as the opportunity to talk without actually wanting to go out (...) I hope it would encourage seeking support more” (Staff 7). Others feared that the change would take away the opportunity for that type of conversation: “For some of the women they don’t always feel able to approach us for one-to-one time but with that risk assessment that would be that protected time” (Staff 8). Moreover, if patients do find that time, it “might overload the nurses if they’re having more of these informal chats all the time” (Staff 9).

The final sub-theme reported by staff was staff-patient conflict. Staff described how common conflict is with the current risk assessment procedure, given that patients see them as the gatekeepers to leave from the ward. They felt that the proposed changes “would definitely reduce the friction” (Staff 9) because they involve the restoration of autonomy to the patient: “The few times there is aggression or aggressive language or behavior, it’s usually around being allowed out, so I think it would lower the risk on the ward” (Staff 7). If patients knew that the decision-making ability was in their hands, “we would possibly have less of patients trying to put the responsibility on staff, so, ‘you let me out so you didn’t care about me,’ that sort of thing” (Staff 7).

On the other hand, the patients might resist the idea of staff offloading the responsibility onto them, thus introducing more conflict: “The situations where the women have been let out (...) without following [the risk assessment] process [have] had a large backlash in terms of sense of trust and caring for them, in their words, ‘we don’t give a shit’” (Staff 8). Alternatively, if staff retain the right to advise a patient to stay on the ward following an optional conversation, the advice may not be welcomed by the patient, so “there’s the potential to return to the similar issue of conflict which we had before” (Staff 2).

Time Taken

A total of seven (88%) patients and five (50%) members of staff felt that risk assessments currently take too long, which impacts negatively upon both parties. All seven patients expressed their frustrations with having to wait for staff to do a risk assessment before they are allowed to go out: “It just takes time and you just wanna go out. It’s not like that in the real world, it’s just time-consuming and inconvenient” (Patient 8). One patient also felt that the time taken for risk assessments can affect her mood: “Waiting for a risk assessment can be very frustrating especially if you are already struggling or you then feel like a burden for the amount of time it uses up which can then negatively impact your mood” (Patient 1). This view was echoed by Staff 9: “Their needs can be met sooner, because sometimes when the shift is busy, you have to ask them to wait which can make them quite frustrated and actually makes them feel worse.” Furthermore, many patients pointed to the fact that they feel as if they are wasting staff’s time: “The current risk assessment process takes such a long time, often people won’t go out because they don’t want to burden staff” (Patient 1). Many staff reaffirmed this point of view when referring to the current process: “A lot of nursing time is wasted” (Staff 7).

In terms of the proposed changes to the process, four patients pointed out that the changes would encourage them to leave the ward more often, preparing them for life outside the ward: “I think it would help improve us getting out into the community. For example, going out for walks or going out to Tesco like we would at home because we would feel less like we’re annoying or a burden” (Patient 1). However, one patient did voice concerns over the fact that, in order to save time, patients may choose not to have a conversation with staff, and instead leave the ward regardless of their mental state: “I think people are less likely to have a chat just in terms of wanting to get out quicker of the ward” (Patient 3). Nevertheless, this patient also felt that with the time saved on not having to do risk assessments, staff would be able to give more time to patients and attend to them better: “I think it’s getting more time for staff to look into what we’re doing” (Patient 3). This view was also held by two members of staff who thought that making the risk assessment process shorter, with less paperwork, would mean that staff “would have more time to be with patients so that could have an impact on listening more” (Staff 7) and “nurses could actually spend more time with patients rather than having their heads glued to a computer” (Staff 9).

Finally, one member of staff (Staff 2) did voice their concerns that the new risk assessment procedure may be seen as “a time-saving measure,” rather than one designed to improve the quality of care provided to patients.

Chance for Reflection

A total of three (38%) patients and one (10%) member of staff talked about the current risk assessment procedure as a chance to reflect before leaving the ward. They thought that patients sometimes discover through having the conversation that they are not in a fit state to leave the ward: “In their mind they think, ‘I’m going to do this,’ and then they have to go in and sit down. I think sometimes it’s a well-timed pause” (Staff 8). Patient 4 felt that “they are needed as a barrier to make you think of whether you are safe to leave, and also to force you to have a chat when you need it but don’t want it.” However, Patient 7 stated that if a patient finds the conversation useful, the proposed change to the risk assessment procedure would still allow her to have that: “For me, what I would do is stick to having a conversation before I leave. That would make me feel more safe. It gives me a chance to self-evaluate, and have someone else on the outside give me an opportunity to talk.”

Triggering Negativity

A total of three (38%) patients but no members of staff felt that the current risk assessment procedure can trigger negative thoughts by forcing them to answer questions about their risk of suicide and self-harm: “You want to go out and when you’re feeling really good, they ask you all these questions about how you’re going to keep yourself safe, but the thought of doing anything that was unsafe hadn’t even crossed your mind so you’re like, ‘well...thanks’” (Patient 8). Patient 5 echoed these sentiments: “I find that being asked if you’re suicidal is degrading and triggers suicidal thoughts.”

Ideas About Risk

Nine (90%) members of staff but no patients discussed the concept of risk as applied to the patient group. First, staff acknowledged that risk is extremely difficult to predict, especially for patients with a personality disorder: “This diagnosis is unpredictable” (Staff 6); “If they’re going to do it, they’ll do it” (Staff 9). Staff 2 mentioned that the patients have a chronic risk of suicidal and self-harming behaviors, whereas most risk assessments are used in acute settings for people with an immediate short-term risk. Furthermore, this individual highlighted that studies have found no evidence for the use of risk assessment tools, rather “there is evidence to support that we shouldn’t use them.”

However, Staff 4 said it is not necessarily about risk prediction but about risk reduction through the development of strong staff-patient relationships. With a view to changing the risk assessment process, this person thought that the level of risk would not change unless strong staff-patient relationships are established, at which point the level of risk would decrease, regardless of the change in procedure. On a similar note, most members of staff viewed the risk assessment process as one that is ongoing and inherent to everything that happens on and off the ward, not as an event confined to the point of a patient requesting leave from the ward: “Mental health nurses are trained to assess risk all the time” (Staff 1); “Part of my role as a nurse is to risk assess (…) [by] interacting with them and chatting to them on a day to day basis” (Staff 8).

Some members of staff took the concept of risk more broadly to include the risk to patients associated with changing the risk assessment procedure. Most welcomed the proposed changes overall, suggesting that “this ward has been successful by making perceived ‘risky’ changes, so why not make this change” (Staff 4); “we’re a really good team in terms of positive risk-taking and I think this would be the next positive step in becoming more of a therapeutic community as opposed to a ward” (Staff 10). However, another thought “this ward is so far removed from any other type of service that potentially that could be a step too far” (Staff 8). Additionally, many staff members felt that risk off the ward would be unaffected by the change to the risk assessment process: “But the risk out in the community would probably be the same” (Staff 7).

## Discussion

Our study demonstrated that the majority of patients and staff on a psychiatric ward were in favor of a more time-efficient risk assessment procedure that gives more responsibility and autonomy to patients, while also encouraging a holistic approach from staff that caters to the unique needs and circumstances of each individual patient. There was overwhelming agreement between staff and patients on all of these major themes, which is in contrast to previous studies on the subject [17]. In keeping with our findings, previous literature has also highlighted mental health staff’s preference for favoring clinical judgment, experience, and instinct in the formation of a holistic risk assessment, as opposed to using risk assessment tools that are seen as bureaucratic by some patients [4,18,19].

Both patients and staff have previously discussed the benefits of involving service users in decisions regarding their care [18,19], which is now recommended by The Royal College of Psychiatrists [20]. Indeed, many staff in our study admitted that they attempt to make the process more person-centered than the “tick-boxey” risk assessment can allow; an effort that is largely appreciated by patients. As such, with regards to the dichotomy of clinical versus actuarial approach that is central to the debate on risk assessments [21], participants in our study favored a clinical approach that values the expertise and knowledge of mental health staff in assessing each individual patient.

The potentially harmful impact of standardized risk assessments was demonstrated by two patients in our study. These individuals expressed concerns about answering questions pertaining to their risk of suicide and self-harm as part of every risk assessment, which could trigger negative thoughts. While several studies have found that suicidal ideation is not increased by inquiring about suicidal thoughts [22], BPD patients have been found to be more sensitive to such questions, with one study finding that 70% of a cohort of female BPD patients reported at least one instance of an increase in suicidal ideation following assessment over a two-year period [23]. Therefore, in a group of inpatients with a chronically high risk of self-inflicted injury or death, it may be questioned whether it is of therapeutic benefit to constantly inquire about patients’ thoughts of suicide and self-harm when these are well-established features of their diagnosis.

Despite the predominant positivity regarding the proposed leave protocol, patients and staff who raised concerns were most troubled with the shift in responsibility associated with it. Especially in an inpatient setting, it can be argued that patients are hospitalized specifically because they cannot keep themselves safe, and it is therefore the staff’s duty to help them with this. If patients can choose to leave the ward at any time, they may be putting themselves in harm’s way. However, as emphasized by both patients and staff, the reliance on staff to keep patients safe has its limitations. The ward is a rehabilitation environment that is designed to help patients return to an independent life within the community after discharge; their admission, with the help of DBT skills, should be spent learning how to keep themselves safe, which is a challenge if staff are held accountable for decisions made by patients. At the very least, a change towards shared responsibility between staff and patients is recommended [24], and for this to work appropriately, it would need to be accompanied by organizational processes that support rather than blame healthcare staff in order to facilitate learning and reflection following adverse events [25].

Sole staff responsibility for patient actions can give rise to risk-averse practices, which, while designed to protect patients and the public, have historically led to repressive conditions for patients [26]. Such practices continue to harm patients to this day as they can invoke the notions of “danger,” suggesting that risk is undesirable [27]. This perception results in an approach centered on risk minimization, which unhelpfully denies patients certain “risky” learning opportunities, such as leave from the ward that may aid their long-term recovery [4]. On a practical level, risk is fundamental to all human activities, regardless of the person’s mental well-being, and it cannot realistically be eliminated altogether [28]. As such, positive risk-taking provides an invaluable contribution to the therapeutic development of psychiatric inpatients [28]. Considering the balance that needs to be sought between the minimization of risk and the promotion of positive risk-taking, it is important to integrate the concept of risk with those of safety and personal recovery [24]. The ward should promote recovery-oriented safety planning that enhances each patient’s “capacity to develop self-directed plans to manage risk in the pursuit of valued life goals” [24].

Naturally, open and honest dialogue between patients and staff is central to such a ward environment. However, patients in our study felt that the traditional risk assessment approach discourages this by encouraging patients to give dishonest responses in order to attain leave from the ward. Evidence from forensic settings points to patients being well-aware of regular monitoring and reporting of their behavior [29], which may limit their capacity for autonomy should their true thoughts and feelings be perceived as risky by staff [17]. Considering the effect that risk perception has on openness and honesty, it could be argued that a risk assessment that determines leave from the ward may not represent a patient’s true mindset and is thus meaningless. Instead, any evaluation of risk should be of limited consequence to the patient’s autonomy so as to encourage a thorough, open, and honest discussion with the patient.

Limitations

One possible limitation of this study was that, as the patients being interviewed had frequent mood fluctuations as a feature of their diagnosis, their state of mind at the time of their interview may have influenced their responses. This may have been partly accounted for by performing a Mental State Examination prior to their interview. However, we decided not to do this as it could come across as judgemental of a patient’s mental state and suggests that opinions are less valid in certain mental states. A further limitation was that one patient and two members of staff were unavailable for an interview. Nevertheless, the participant samples were large in proportion to the ward population. The potential influence of an interviewer-participant power dynamic may have been an additional limitation. However, each participant was aware that as the interviewers were medical students, they had no impact on patient care. Finally, the structured interviews may have limited the range of themes that were elicited. However, we regularly used the open question “why?” to allow each participant to expand on their thoughts and feelings regarding each question, and we did not apply any limits to what patients could say in response.

## Conclusions

Ultimately, all mental health wards designed for long-term admission must strike a balance between protecting patients and preparing them for life after hospitalization. In our study, we reported the views of patients and staff on two different types of risk assessment procedures, finding there to be considerable agreement between these participant groups. The majority favored a risk assessment procedure that would grant patients more freedom and responsibility, while encouraging staff to individualize the process for each patient by taking a holistic approach. The proposed leave protocol, which through this study has given staff and patients the opportunity to express their views, is one step in the direction towards a ward environment that is more recovery-focused and less risk-averse. We will report the outcomes of using the new risk assessment in a separate article.

## Appendices

Outline of the interview structure

Questions regarding the preexisting risk assessment procedure

How satisfied are you with the current risk assessment process on a scale from 1 to 5 where 5 is the most satisfied you could be? Why? If you could make your own risk assessment process, what would it look like?

Explanation of the new leave protocol

When you want to leave the ward, you would be asked whether you want to have a conversation first. If you say you don’t want to talk, then you would be free to leave. If you say you do want to talk, then you would have that conversation with your 1:1 or any other member of staff and they might advise you on whether to leave the ward or not, but YOU would make the final decision whether to leave the ward. The reasons why this has been suggested are: 1. It would reduce friction between staff and patients 2. It would promote patient responsibility for their own safety 3. It would feel less like a hospital 4. It would encourage patients to ask for help when needed 5. It would reduce time. Any questions?

An equivalent version with the necessary grammatical changes was read out to interviewed staff.

Questions regarding the new “leave protocol”

Does this process sound safer, less safe, or just as safe as the current process? Why? Does it promote more independence, less independence, or the same? Why? Do you think you would feel more responsible for your own health, less responsible, or the same? Why? Do you think you would listen to staff more, less, or the same? Why? Do you think you would be more likely to engage in risky behaviors, less likely, or the same? Why? Do you think you would seek help from staff more, less, or the same? Why? Do you have any other comments?

The same questions were asked to the staff, with the necessary grammatical changes to make the questions refer to the patients.
